# Malaria risk factors in Butajira area, south-central Ethiopia: a multilevel analysis

**DOI:** 10.1186/1475-2875-12-273

**Published:** 2013-08-02

**Authors:** Adugna Woyessa, Wakgari Deressa, Ahmed Ali, Bernt Lindtjørn

**Affiliations:** 1 Ethiopian Health and Nutrition Research Institute, P. O. Box 1242/5654, Addis Ababa, Ethiopia; 2 School of Public Health, College of Health Sciences, Addis Ababa University, P.O. Box 9086, Addis Ababa, Ethiopia; 3 Centre for International Health, University of Bergen, Bergen, Norway

**Keywords:** Malaria risk, Multilevel analysis, Highland-fringe, Butajira area, Ethiopia

## Abstract

**Background:**

The highlands of Ethiopia, situated between 1,500 and 2,500 m above sea level, experienced severe malaria epidemics. Despite the intensive control attempts, underway since 2005 and followed by an initial decline, the disease remained a major public health concern. The aim of this study was to identify malaria risk factors in highland-fringe south-central Ethiopia.

**Methods:**

This study was conducted in six rural kebeles of Butajira area located 130 km south of Addis Ababa, which are part of demographic surveillance site in Meskan and Mareko Districts, Ethiopia. Using a multistage sampling technique 750 households was sampled to obtain the 3,398 people, the estimated sample size for this study. Six repeated cross-sectional surveys were conducted from October 2008 to June 2010. Multilevel, mixed-effects logistic regression models fitted to *Plasmodium* infection status (positive or negative) and six variables. Both fixed- and random-effects differences in malaria infection were estimated using median odds ratio and interval odds ratio 80%. The odds ratios and 95% confidence intervals were used to estimate the strength of association.

**Results:**

Overall, 19,207 individuals were sampled in six surveys (median and inter-quartile range value three). Six of the five variables had about two-fold to eight-fold increase in prevalence of malaria. Furthermore, among these variables, October-November survey seasons of both during 2008 and 2009 were strongly associated with increased prevalence of malaria infection. Children aged below five years (adjusted OR= 3.62) and children aged five to nine years (adj. OR= 3.39), low altitude (adj. OR= 5.22), mid-level altitude (adj. OR= 3.80), houses with holes (adj. OR= 1.59), survey seasons such as October-November 2008 (adj. OR= 7.84), January-February 2009 (adj. OR= 2.33), June-July 2009 (adj. OR=3.83), October-November 2009 (adj. OR= 7.71), and January-February 2010 (adj. OR= 3.05) were associated with increased malaria infection.

The estimates of cluster variances revealed differences in malaria infection. The village-level intercept variance for the individual-level predictor (0.71 [95% CI: 0.28-1.82]; SE=0.34) and final (0.034, [95% CI: 0.002-0.615]; SE=0.05) were lower than that of empty (0.80, [95% CI: 0.32-2.01]; SE=0.21).

**Conclusion:**

Malaria control efforts in highland fringes must prioritize children below ten years in designing transmission reduction of malaria elimination strategy.

## Background

About half of the total population living between altitudes of 1,500 and 2,500 m above sea level (masl) is at risk of malaria and the areas experience epidemics in Ethiopia [1]. Recent studies from high-altitude areas identified age, nearness of houses to breeding places, sharing of houses with animals, presence of windows and open eaves as malaria risk factors [2-6]. Moreover, malaria is associated with environmental factors such as altitude, rainfall, and temperature [7,8]. Thus, malaria interventions target both households and environment. This necessitates use of multilevel analysis to identify malaria risk factors at individual or household levels. Identification of malaria risk factors at different levels, including at ecological level, is helpful in designing targeted interventions of malaria control measures [9]. However, only one study done in Adama Town considered individual- and household-level malaria risk factors using multilevel analysis [10].

Since 2005, Ethiopia has scaled up malaria control programs using key malaria interventions such as effective case management (artemisinin combination therapy and malaria rapid diagnostic tests), and vector control options (indoor residual spray and long-lasting insecticidal nets) in endemic areas (<2,000 masl). Subsequently, the program obtained fruitful results in reducing malaria burden between 2006 and 2008. In addition, the 2011–2015 National Strategic Plan highlights the intent to eliminate malaria in specific geographical areas with historically low malaria transmission; and achieve near zero malaria deaths in the remaining malarious areas of the country [11]. Thus, understanding the epidemiology of highland malaria, which is considered as at high epidemic risk is vital in improving malaria control efforts, and furthermore successfully eliminate malaria. A recent study described prevalence of malaria influenced by altitudinal location of households, survey season and age of participants [12]. Prevalence increased from high to low altitude that also revealed differences at varying age categories. In addition, this study confirmed seasonality of malaria. Malaria endemic areas that show a large variation from one year to another in the number of malaria cases considered as high epidemic risk. Thus, a present study area is considered as at high risk of epidemic malaria. A study was aimed at estimating malaria prevalence using longitudinal data illustrated seasonal variation of malaria infection. Moreover, prevalence of malaria differed by about 20-fold between villages of low prevalence and high prevalence. More interestingly, prevalence varied with different age categories along three altitudinal strata [12].

Accordingly, identifying factors influencing malaria infection both at individual-and village-levels (clusters of household) appears useful in guiding targeted malaria interventions at highland areas with malaria low prevalence and at high epidemic risk. In addition, the Ministry of Health of Ethiopia recommended evidence-informed decision to incorporate high altitude areas (>2,000 masl) in malaria control program [13]. Thus, understanding the local malaria epidemiology in small areas with altitudinal differences like Butajira area is helpful in improving malaria interventions at high altitudes. Thus, the purpose of this study was to generate community-based prospective data to examine malaria risk factors in highland and highland-fringe areas. The objective of this study was to identify malaria risk factors using multilevel analysis at highlands of southern Ethiopia.

## Methods

### Study area and study participants

**Figure 1:**
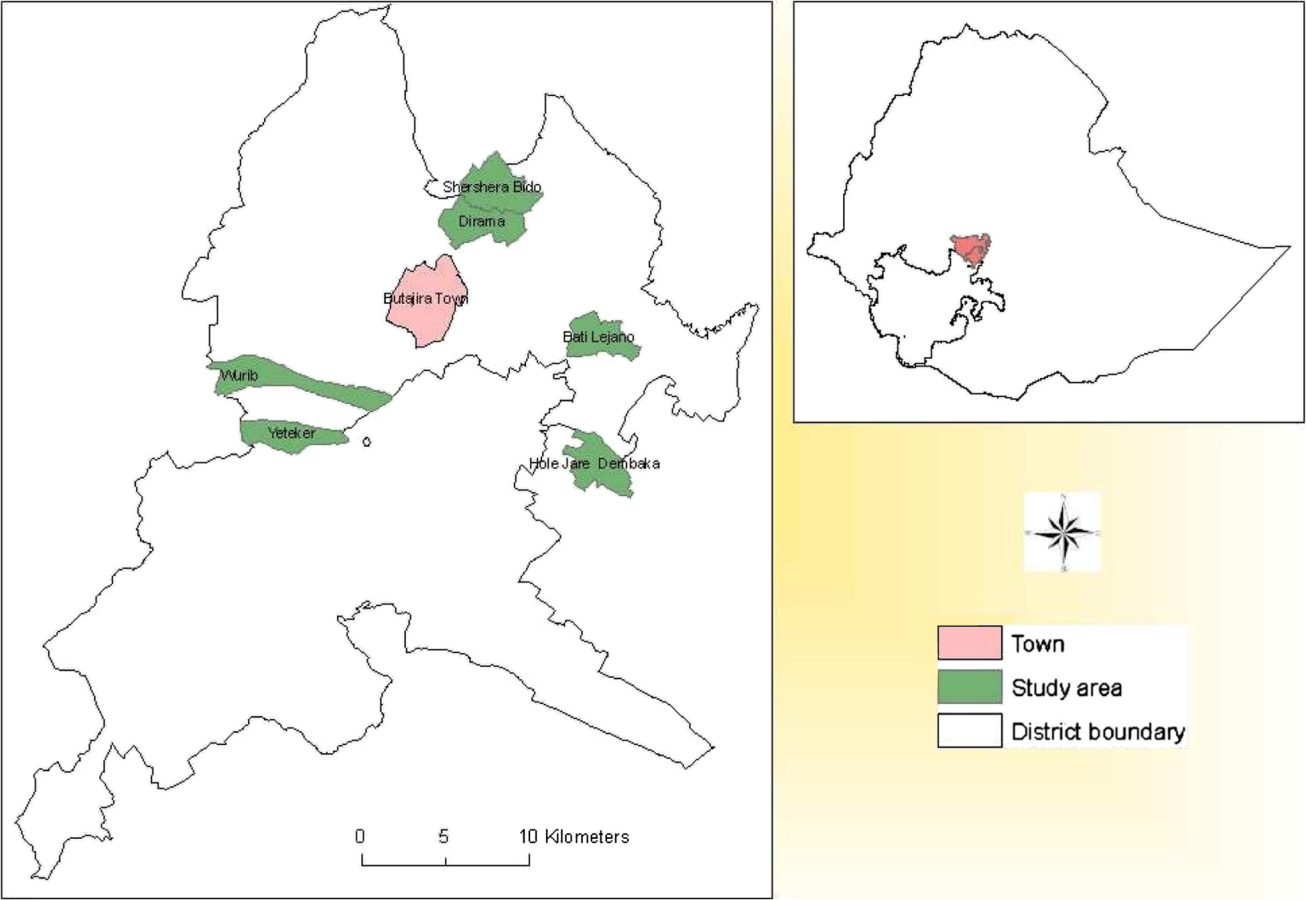
Location of the study sites, Butajira area, South-central Ethiopia.

This study was conducted in six rural *kebeles* (the smallest administrative units) in Butajira area using the demographic surveillance system site at Butajira Rural Health Programme (BRHP) [14], located about 130 km south of Addis Ababa. The study area is administratively located in Meskan and Mareko districts. These districts are found in Guraghe Zone, Southern Nations Nationalities and Peoples (SNNP) Regional State of Ethiopia (Figure 1). There were 58,335 people living in the BRHP DSS in 2008. Half (50.1%, n=29,243) of the population were females. About half of the total population (46%, n=26,834) of the people lived in the study areas. Most people in the area practice subsistence farming. The study area is part of an altitudinal transect between 1,800 and 2,300 masl. The mean annual rainfall of the study area during the last nine years was 945 mm (yearly range 510 mm to 1,329 mm). However, annual rainfall was below the average in 2009 and 2010. The main rainy season is usually from June to September. The mean temperature was 18.2 degrees, with average annual minimum and maximum temperature of 10.0 and 26.3 degrees Celsius (°C), respectively. The study was conducted is located in the temperate areas.

Malaria is one of the important causes of sicknesses in Butajira area. Between 2004 September and 2010 August, 32.3% (19,923 of 61,654) were microscopically confirmed malaria cases from Butajira and Enseno Health Centres, and Butajira Hospital. On average, more than 10 thousand malaria suspected cases visited these public health facilities between 2002 and 2010. Indoor residual spraying (IRS) operation was performed mainly for epidemic control in the low altitude areas of the present study area. During 2009/2010, a spraying of houses was done to control malaria outbreak in Hobe and Bati Lejano *kebele*s. A recent study found a very low household ITN ownership (28.5%, 171 of 739) with the higher proportion at low (54.2%) than high (3.5%) altitudes. Most of those households (83%, n=142) had at least a family member who slept under a net the night *prior* to the survey [15].

### Sample size calculation

The sample size required for this study was estimated as follows. Estimation of the sample size for malaria prevalence was based on 4.1% prevalence from three Ethiopian regions. The study used a sample size estimated (n=3,398) to measure malaria prevalence in this study area [12]; this sample size was considered adequate to assess risk factors of malaria infection in Butajira area. Using assumptions of expected prevalence of 4%, margin of error =1%, α=5% (95% confidence level), design effect =2 and 15% non-response rate, a sample size of 3,393 people was calculated. Thus, with the assumption of 4.5, average family size of 3,393 people were recruited from 750 households in 16 villages.

### Study design and sampling procedure

This study used community-based repeated cross-sectional survey. Six rural *kebele*s (Hobe, Bati Lejano, Dirama, Shershera Bido, Yeteker and Wurib), two from each of the three different altitudinal strata, were selected. These strata were low, mid-level, and high altitude areas. These localities with varying altitude were found to be suitable for the present study. The target of our survey was 4,816 households residing in those six *kebele*s. Sixteen villages (or clusters of households) with 750 households were randomly selected from the six *kebele*s for the surveys using probability proportion to size (PPS) sampling method. Since it was expected that lower malaria prevalence in the highlands, proportionally selected more households from the highlands. Six cross-sectional surveys were done on the same households for two consecutive years. Blood specimens were collected from October 2008 to June 2010 on a quarterly basis. The surveys were conducted in October-November 2008 (a month after the main rainy season), in January-February 2009 (dry season), in June-July 2009 (main rainy season), in October-November 2009, in January-February 2010, and in June 2010.

### Data collection

This study obtained data from household head interviews of sampled households and blood film collection and examinations of family members. Trained data collectors conducted the interviews using a pretested and structured questionnaire to obtain baseline socio-demographic and household characteristics. Standard procedures were followed for blood specimen collection, processing, microscopic examination and reporting of malaria parasites [16]. Thin and thick films were prepared and Giemsa-stained. Thin films were fixed with alcohol, and both thin and thick films were stained with 3% Giemsa-stain for 30 minutes. Microscopic examination was performed at 1,000x magnification. Negative slide results declared after 100 fields had been thoroughly examined without obtaining *Plasmodium* parasites.

All positive slides and 10% of slides with negative results were sent to another microscopist blinded to the microscopy results to ensure quality of light microscopy. To ensure maximum response rate of participants, households with absentees were revisited once more. The altitude readings of the sample households were recorded using hand-held Global Positioning System (GPS) (Garmin eTrex ®). The principal investigator (AW) and two data collectors (postgraduate students from Addis Ababa University) conducted the GPS recording.

### Calculation of household wealth index

This study used a dataset of relative household wealth index data computed in another study [15]. The procedure for calculating this index is briefly presented as follows. SPSS software was used to perform Principal Component Analysis (PCA) to construct the relative household wealth index, as previously recommended [17], and other similar studies also applied the same procedure in Ethiopia [3,8]. In these studies, ownership of household assets, type of usual water sources, type of product, and house construction material were used to build the wealth index as input to PCA. In the present study, household asset included land, cow, truck, mill, sewing machine, fridge, television, electricity line, telephone line, and kerosene. Types of products were wheat and barley, *Teff* (*Eragrostis tef*), pepper, Enset (*Ensete ventricosum*), and *Khat* (*Catha edulis*). House construction materials were types of roofing and presence of window.

Suitability of the data for factor analysis was assessed before performing PCA. Thus, both the Kaiser-Meyer-Oklin (KMO) value and Bartlett’s Test of Sphericity results were supporting the factorability of the correlation matrix. PCA result was repeated with alterations until the resulting model was suitable for the survey data. Finally, 11 indicators were selected to run the final PCA. PCA revealed the presence of two components with eigenvalues above 1, explaining 36.0% and 9.8% variance in the dataset, respectively. The first principal component (with eigenvalues of 5.04) represented 36.0% of the variance in the sample and was used to generate the wealth index of the study households.

The 11 variables with greatest weights were loaded on the first principal component: possession of motorcycle (0.937), sewing machine (0.869), truck (0.869), television (0.809), grain-mill (0.646), lantern-kerosene (0.610), phone (0.575), electricity line (0.568), bicycle (0.423), types of sleeping places (0.356) and cart (0.355). The wealth index varied from −0.256 to 13.27. Then, all households were ordered into three wealth groups: the “lowest” ranked group (30.9%, n=228), followed by the “middle” ranked group (35.7%, n=264), and finally the top third in the “higher” ranked group (33.4%, n=247). Data collectors assessed physical condition of houses and recorded the data. Training manual was distributed to help in categorizing the houses into three including dilapidated, houses with their walls allowing mosquito entry and good condition.

### Outcome and predictor variables

Finding *Plasmodium* parasites in blood films upon microscopic examination was the primary outcome of this study. The results expressed as parasite prevalence, i.e. the percentage of *Plasmodium* positive subjects. The factors used to explain outcomes are defined at two levels: individual (age, gender) and village (altitudinal strata, wealth status, house status, and survey seasons).

Variables were categorized and coded as follows. Age grouped into four classes and coded as <5, 5–9, 10–14, and ≥15 years. Altitudinal location of households was classified into three including low (1,800-1,899 m), mid-level (1,900-1,999 m), and high (2,000-2,300 m) altitudes. There were three wealth groups including lowest, middle, and higher; and three house status categories such as dilapidated, walls with holes, and good condition. The survey seasons are October-November 2008, January-February 2009, June-July 2009, October-November 2009, January-February 2010, and June 2010. In all cases the last categories were considered as reference groups.

### Data management and analysis

Data entry and cleaning was done using Epi Info version 6 (Centers for Disease Control and Prevention (CDC), Atlanta, Georgia (USA). Descriptive statics was performed using IBM® SPSS® Statistics version 20.0. Descriptive statistics was performed to describe characteristics of predictor variables. Multicollinearity was checked using linear regression as recommended and no multicollinearity was evident. Multivariate analysis was done using STATA version 11.0 (College Station, Texas, USA). Mixed-effects logistic regression was fitted using selected independent variables to estimate individual *Plasmodium* infection, the outcome variable. This model computes the regression using a two-stage system of equations, which involves the lowest level and higher level equations. In this case, individual as the lowest level that explains the individual variation within each village; and the village-level that explains variation across villages were entertained as suggested [18].

Multilevel analysis is a statistical tool applied to data with nested sources of variability, which involve units at lower level nested within units at a higher level [19]. Moreover, this statistical tool allows the simultaneous examination of the effects of group level and individual level variables on individual level outcomes while accounting for the non-independence of observations within groups. Similarly, previous malaria epidemiological studies have also applied multilevel analysis to identify malaria risk factors [9,10]. In this study, both univariate and multivariate multilevel modelling was performed using mixed-effects logistic regression. In univariate multilevel, the relationship of each variable through entering village, higher-group, was analysed. Then, multivariate multilevel modelling was performed in a stepwise process in three steps: the first step examined the empty model, i.e., without adjusting for predictors; the second step included individual-level predictors with village-level; the final model was fitted using village-level and individual-level predictors identified as significant in the second step, to obtain the full model.

Intra-class correlation coefficients (ICC), median odds ratios (MOR) and 80% interval OR (IOR-80) were computed to estimate village-level variance in *Plasmodium* infection. MOR and IOR-80 are recommended for estimating variability of binary outcomes to overcome contextual and interpretational problems with ICC in a binary outcome [18]. In multilevel logistic regression, the individual level variance and the area level variance are not directly comparable unlike in the case of multilevel linear regression. Thus, some methods were used to convert the individual level and area level components of the variance to the same scale before computing the ICC [18]. This study used *latent variable method* that converts the individual level variance from the probability scale to the logistic scale, on which the area level variance is expressed. Thus, in this study, the method assumes that the propensity for malaria infection detected is a continuous latent variable underlying the binary outcome (that is, having malaria infection or not). This meant, every person has a certain propensity for getting malaria infection but only persons whose propensity crosses a certain threshold detected as *Plasmodium* positive. The unobserved individual variable follows a logistic distribution with individual level variance equals to π^2^/3 (i.e., 3.29) [19]. However, the interpretation of the ICC for binary outcomes remains difficult to understand in epidemiological terms due to inherent statistical consistency of ICC [20].

MOR was computed to translate the area level variance in the widely used odds ratio scale. The MOR is defined as the median value of the odds ratio between the area at higher risk and the area at lowest risk when randomly picking out two areas the MOR can be conceptualised as the increased risk that (in median) would have if moving to another area with a higher risk [18]. The MOR is statistically independent of the prevalence of the phenomenon, and can be easily computed in the empty model and in more elaborated models. Regarding the interpretation of MOR, if the MOR is equal to one, there would be no differences between areas in the probability of getting malaria infection while if there were strong area level differences, the MOR would be large and the area of residence would be relevant for understanding variations of the individual probability of malaria infection detected. The standard error (SE) of the area level variance indicates the precision of the estimate. Since MOR quantifies group-level variance in terms of odds ratios, it is comparable to the fixed effects odds ratio, which is the most widely used measure of effect for dichotomous outcomes.

In order to integrate the area level fixed effect and the random residual variations using the 80% interval odds ratio (IOR-80%) is suggested [18]. In interpreting the IOR-80, the interval is narrow if the residual variation between areas is small, and wide if the variation between areas is large. If the interval contains the value one, this indicates that the effect of the area characteristic under investigation is not that strong when compared with the remaining residual area level heterogeneity. The percentage of proportional change in variance (PCV) was calculated as presented [18]. Adjusted odds ratios with 95% confidence intervals and standard errors obtained from regression coefficients were used to assess the associations of the predictors and outcome variable. Statistical significance was considered at *p*<0.05.

### Assessing models

To assess whether a model predict the outcome variable beyond what would be expected by chance, the familiar Chi-Square likelihood-ratio test of a difference between models is used. The degrees of freedom for the Chi-Square are the differences in the number of parameters for the models being compared [21].

In this study, individual-level predictor model (with −2 Log Likelihood value of 969.7091 and 4 df) compared against the full model (with −2 Log Likelihood value of 886.97871 and 15 df). By subtracting, the difference (969.7091 and 886.97871) is 55.9 and this value showed statistically significant with (15–4) = 9 df, so the full model leads to prediction that is significantly better than chance.

### Ethical consideration

Ethical approval of the study was obtained from the Faculty of Medicine at Addis Ababa University, and the Ethiopian Ministry of Science and Technology. Individual informed consent was obtained from adults, and from the parents or guardians of children aged less than 18 years. In addition, minors gave verbal assent. Blood specimens were collected as recommended using an alcohol swab and disposable blood lancets by trained staff [16]. All people found to be malaria positive during the survey were treated according to the national guide line [22].

## Results

### Characteristics of study participants

**Figure 2:**
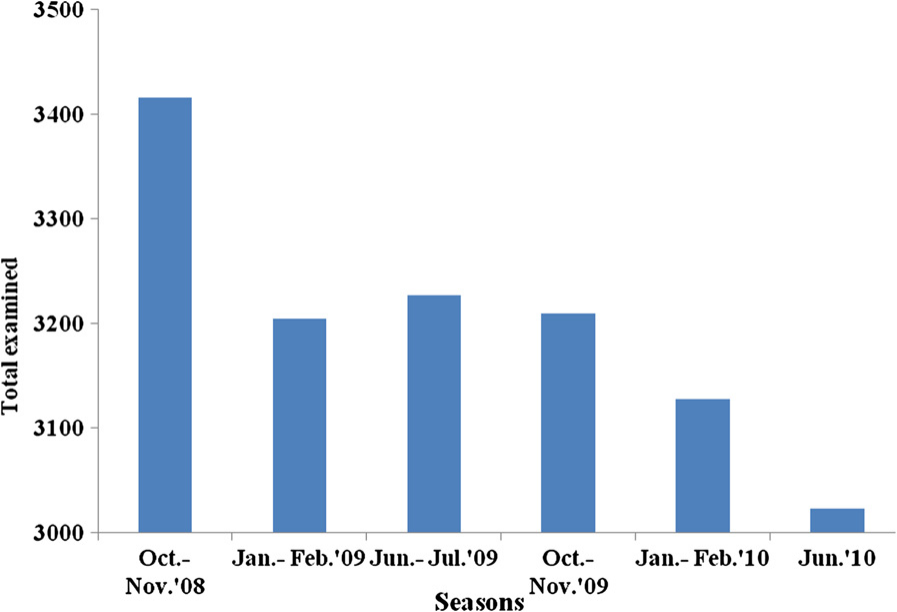
The number of study participants in baseline and follow-up surveys, Butajira area, Ethiopia, 2008–2010.

Overall, 19,207 individuals were sampled in six surveys (median and interquartile range value of three). Most of the participants were 15 years old and above with a mean (±SD) age of 20.5 (±17.2), and the range was between one month and 99 years. Above half (51.3%) of the participants were females. A total of 3,416 participants were included in the baseline survey conducted during October-November 2008. In the consecutive five follow-up visits, there were 3,205 (January-February 2009), 3,227 (June-July 2009), 3,210 (October-November 2009), 3,127 (January-February 2010), and 3,022 (June 2010) participants sampled (Figure 2).

A recent study estimated 0.93% (178 of 19,207) of the participants had malaria infection. Most of the infections were due to *Plasmodium vivax* (86.5%, n=154) and the rest due to *Plasmodium falciparum* (12.4%, n=22) and mixed infections (1.1%, n=2) [12]. Prevalence of malaria infection varied with age of participants, season, and household factors such as altitudinal location, wealth status, and housing conditions. Increased malaria prevalence was observed in children aged below five and five to nine years, low altitude, mid-level altitude, low wealth status and in houses having holes in their walls (Table 1).

**Table 1:** **Prevalence of *Plasmodium *infection in Butajira area, Ethiopia, October 2008 to June 2010**

**Factors**	**Total examined, n**	**Positive, n (%)**	**Chi-Square**	***P*-value**
***Individual***				
**Total**	**19,207**	**178 (0.93)**		
**Age category**			70.8	<0.001
<5	3,042	54 (1.77)		
5-9	3,513	59 (1.68)		
10-14	2,702	20 (0.74)		
≥15	9,942	45 (0.45)		
**Gender**			3.4	0.06
Male	9,347	99 (1.06)		
Female	9,852	79 (0.80)		
***Household***				
**Total**	**738**	**44 (6.00)**		
**Wealth status**			70.0	<0.001
Lowest	6,379	111 (1.74)		
Middle	6,419	40 (0.62)		
Higher	6,401	27 (0.42)		
**House status**			39.0	<0.001
Dilapidated	3,195	21 (0.66)		
Holes	4,844	81 (1.67)		
Good	11,160	76 (0.68)		
***Village***				
**Altitudinal strata**			106.5	<0.001
1,800-1,899 m	5,547	107 (1.93)		
1,900-1,999 m	2,034	29 (1.43)		
2,000-2,300 m	11,618	42 (0.36)		
**Seasons**			86.5	<0.001
October-November 2008	3,416	20 (0.58)		
January.-February 2009	3,205	11 (0.34)		
June-July 2009	3,227	27 (0.84)		
October-November 2009	3,210	72 (2.24)		
January.-February 2010	3,127	35 (1.11)		
June 2010	3,022	13 (0.43)		

### Univariate analyses

In the univariate logistic regression, children aged below five years (unadjusted OR= 3.71), children aged five to nine years (unadj. OR= 3.40), low altitude (unadj. OR= 5.12), mid-level altitude (unadj. OR= 3.63) and houses with holes (unadj. OR= 1.57) had increased risk of having *Plasmodium* infection. Similarly, survey seasons including October-November 2008 (unadj. OR=7.95), January-February 2009 (unadj. OR=2.35), June-July 2009 (unadj. OR=3.76), October-November 2009 (unadj. OR=7.68), and January-February 2010 (unadj. OR=2.93) showed higher malaria prevalence (Table 2).

**Table 2:** Predictors of malaria risk obtained from mixed-effects logistic regression analysis, Butajira area, Ethiopia, October 2008 to June 2010

**Fixed-effects**		**Unadj. OR (95% CI)**	**Adj. OR (95% CI)**
**Age groups**			
<5		3.71 (2.49-5.52)**	3.62 (2.43-5. 40)**
5-9		3.40 (2.30-5.02)**	3.39 (2.30-5.01)**
10-14		1.48 (0.87-2.51)	1.49 (0.88-2.53)
**Gender**			
Male		1.33 (0.99-1.79)	1.24 (0.92-1.67)
**Altitudinal strata**			
1,800-1,899 m		5.12 (3.29-7.98)**	5.22 (2. 67–10.22)**
IOR-80%			(3.74-7.24)
1,900-1,999 m		3. 63 (2.02-6.52)**	3.80 (2.09-6.91)**
IOR-80%			(2.72-5.26)
**Wealth group**			
Lowest		2.02 (1.03-3.94)	0.75 (0.37-1.53)
Middle		1.32 (0.78-2.22)	1.00 (0.59-1.69)
**House status**			
Dilapidated		1.00 (0.60-1.66)	1.00 (0.60-1.67)
Holes		1.57 (1.11-2.22)*	1.59 (1.12-2.26)*
IOR-80%			(1.14-2.22)
**Seasons**			
October-November 2008		7.95 (3.94-16.01)**	7.84 (3.89-15.81)**
IOR-80%			(5.64-10.91)
January.-February 2009		2.35 (1.05-5.25)*	2.33 (1.04-5.21)*
IOR-80%			(1.68-3.16)
June-July 2009		3.76 (1.94-7.30)**	3.83 (1.97-7.43)**
IOR-80%			(2.74-5.31)
October-November 2009		7.68 (4.25-13.88)**	7.71 (4.26-13.93)**
IOR-80%			(5.53-10.70)
January.-February 2010		2.93 (1.54-5.54)*	3.05 (1.61-5.77)*
IOR-80%			(2.18-4.22)
**Parameters/Models**	Empty [95%CI] (SE)	Individual-predictor [95%CI] (SE)	Final [95%CI] (SE)
**Fixed-effects**			
Village intercept	0.81 [0.49-1.31] (0.25)	0.11 [0.06-0.18] (0.28)	0.01 [0.006-0.03] (0.37)
Village intercept variance	0.80 [0.32-2.01] (0.21)	0. 71 [0.28-1.82] (0.34)	0.034 [0.002-0.615] (0.05)
**Random- effects**			
ICC (%)	19.5	17.7	1.0
MOR	2.34 (0.21)	2.23 (0.34)	1.19 (0.05)
PCV (%)	-	11.2	95.7

*P<0.05, ** *P*<0.001, IOR-80=interval odds ratio-80, *SE*, standard error of variance,

*ICC*, intra-class correlation co-efficient, *MOR*, median odds ratio, *PCV*, proportional change variance.

### Multivariate, multilevel models

Multilevel logistic regression of the fixed effects showed that age, altitudinal location, house status, and seasons were related to higher risk of malaria infection. These variables had about two-fold to eight-fold increase in prevalence of malaria. Furthermore, among these variables, October-November survey seasons of both during 2008 and 2009 were strongly associated with increased prevalence of malaria infection (Table 2). Children aged below five years (adjusted OR= 3.62) and children aged five to nine (adj. OR= 3.39), low altitude (adj. OR= 5.22), mid-level altitude (adj. OR= 3.80), houses with holes (adj. OR= 1.59), survey seasons such as October-November 2008 (adj. OR= 7.84), January-February 2009 (adj. OR= 2.33), June-July 2009 (adj. OR=3.83), October-November 2009 (adj. OR= 7.71), and January-February 2010 (adj. OR= 3.05) were associated with increased malaria infection.

The estimates of cluster variances (or random effects) revealed differences in malaria infection. The village-level intercept variance for the individual-level predictor (0.71 [95% CI: 0.28-1.82]; SE=0.34) and final (0.034, [95% CI: 0.002-0.615]; SE=0.05) were lower than that of empty (0.80, [95% CI: 0.32-2.01]; SE=0.21). The ICC value for the final model was 95.7%. Moreover, the MOR values for the empty (2.34±0.21), individual-level predictors (2.23±0.0.34) and final (1.19±0.0.05) models were large (Table 2). This implies that the area of residence (or village) is helpful in understanding variation in malaria infection s of individual.

## Discussion

This study reflects that malaria transmission is highly seasonal and influenced by age of children and altitudinal location as well as poor housing condition at highland-fringe area in rural setting of south-central Ethiopia. The months preceded by main rainy season is found to be consistently a good predictor of increased malaria infection in the present longitudinal study. Multilevel mixed-effects logistic regression analysis found increased malaria risk in children aged below five years, five to nine years, low altitude, mid-level altitude, poor housing condition, and survey seasons in Butajira area, Ethiopia. Most of the cluster-level variance was explained and strong enough using the variables measured. More interestingly, the present community-based longitudinal survey revealed seasonality of malaria transmission that overlapped with abnormal weather condition such as below average annual rainfall. However, health facility-based past studies found an initial reduction of malaria burden following the large-scale interventions in progress since 2005 [23,24]. The occurrence of increased malaria infection especially in younger children following the intensive interventions according to the national strategic plan in endemic areas might require more explanation.

This study has got some limitations. The present study used longitudinal parasitological data for the study participants. However, concurrent ITN possession data was limited to household level and baseline survey period. Household spray status was also incomplete. In both situations the absence of complete data on vector control can be considered as setback of this study. In the present multilevel analysis, household data was aggregated to village-level, which is believed to unnecessarily introduce statistical problem. As missing to follow-up is an inherent problem in longitudinal study, this study might also suffer from this problem. Despite these shortcomings, the present study has contributed to improving sampling problems in which some of the studies have focused on peak malaria transmission season and missed the rest of the seasons. In contrast, this study employed repeated cross-sectional surveys during various seasons with different prevalence. This study also recruited more participants from high altitudes with expected low prevalence to increase the probability of finding malaria positives.

The finding of more malaria infection in children aged five to nine years is consistent with a study conducted in highlands of Ethiopia, Kenya and Uganda [10,25,26]. However, this result is in contrast with a study performed in low-transmission setting in Ethiopia and elsewhere [27,28]. The finding of increased malaria infection in low and mid-level altitudes, adjacent to malaria transmission cut-off area, is in harmony with studies from highlands [25,29]. The more malaria infection in houses with walls having holes is consistent with results of another study [30]. The information illustrating larger MOR in all three models reflects that village-level grouping is important to understand variations of the risk of malaria infection, which is in line with a study conducted elsewhere in Ethiopia and Madagascar [9,10].

It is not clear why age-dependent malaria risk is observed in such low-endemic highlands such as Butajira area, where malaria risk is expected to be uniform across all age groups. Obviously, children aged below five years are vulnerable to malaria infection. The possible explanations could be lowest vector control coverage including low insecticide-treated bed net (ITN) coverage and poor ITN conditions in the study area [15]. Other studies also showed very low ITN coverage, which is associated with higher malaria risk in Uganda [31], low ITN use among school-aged children was also reported [32,33]. The decline of malaria burden was suggested as due to shifting of malaria prevalence to children of older age through delaying the age of first infection [34]. The other possible explanation could be the overlapping of different activities of children and *Anopheles* biting behaviour [10].

The finding of high malaria risk in low altitude can be explained by the presence of suitable high ambient temperature and topography that favours mosquito abundance [35,36]. A study found malaria transmission in western Kenya highland is primarily confined to the valley bottom [37]. The low altitude area is suitable for mosquito breeding due to its flat terrain that allows collection of temporary water following rainy season as described in detail in another study in the same study area [12]. Rivers and streams originating from high altitude as well as ponds created after cessation of rain season make their end at both low and mid-level altitudes [12].

The increased risk of malaria in houses with their walls having holes can be due to increased access of mosquitoes to bite humans. Housing conditions allowing mosquito entrance were indicated as malaria risk factors [5,38]. Another study also found association of more malaria infection with poor quality of housing [39]. The geographical location of residence was found important in recognizing differences in malaria infection in individuals. Thus, houses in the same village might share common mosquito breeding places.

In conclusion, this finding showed increased malaria infection is associated with survey seasons, age of participants, altitudinal location and housing conditions in highland-fringe areas with low transmission settings. The current malaria control efforts could benefit through application of targeted interventions to villages of high malaria cases by prioritizing children aged below ten years in highland-fringe areas of low endemicity. Subsequently, seasonal transmission reduction could be operational in low transmission like Butajira area. Future studies should consider designing more frequent observations and incorporate household spray status and ITN use concurrently.

## Competing interests

The authors declare that they have no competing interests.

## Authors’ contributions

AW contributed to conception and design, acquisition of data, analysis and interpretation of data, and drafting the manuscript. WD, AA and BL substantially contributed to conception and design of the study and reviewing the manuscript, revisiting it critically for important intellectual content. BL, AA, WD and AW reviewed the paper and all authors approved the final version.
